# Supplementary material for: Enhanced joint impact of western hemispheric precursors increases extreme El Niño frequency under greenhouse warming
Source: Nat Commun. 2023 Oct 11;14:6356. doi: 10.1038/s41467-023-42115-7 (PMC10567822; doi:10.1038/s41467-023-42115-7)
Supplement: Supplementary file 1 — Supplementary Information [file 41467_2023_42115_MOESM1_ESM.docx]

Supplementary Information

**Enhanced joint impact of western hemispheric precursors increases extreme El Niño frequency under greenhouse warming**

Hyun-Su Jo^1^ and Yoo-Geun Ham*^1^

*^1^Department of Oceanography, Chonnam National University, Gwangju, South Korea*

**Corresponding authors:** Prof. Yoo-Geun Ham, Chonnam National University, Gwangju, Republic of Korea (E-mail: [ygham@chonnam.ac.kr](mailto:ygham@chonnam.ac.kr))

**Table S1. Information of the CMIP5 and CMIP6 models used in this study.** Names of CMIP5 and CMIP6 models, the associated institutions and countries, and the ensemble members used in this study (r1i1p1 in CMIP5 and mostly r1i1p1f1 for CMIP6, with different ensembles labeled in bold).

| **CMIP5 Model** | **Institute, Country** | **Ensemble** |
| --- | --- | --- |
| ACCESS-0 | CSIRO/BOM, Australia | r1i1p1 |
| CCSM4  CESM1-BGC  CESM1-CAM5 | NCAR, USA | r1i1p1 |
| CMCC-CM  CMCC-CMS | CMCC, Italy | r1i1p1 |
| CNRM-CM5 | CNRM, France | r1i1p1 |
| CSIRO-Mk3-6-0 | CSIRO-QCCCE, Australia | r1i1p1 |
| EC-EARTH | Europe-wide consortium | r1i1p1 |
| FIO-ESM | FIO, China | r1i1p1 |
| GFDL-ESM2M | NOAA-GFDL, USA | r1i1p1 |
| GISS-E2-H  GISS-E2-H-CC | NASA/GISS, USA | r1i1p1 |
| HadGEM2-AO  HadGEM2-ES | MOHC, UK | r1i1p1 |
| IPSL-CM5A-LR | IPSL, France | r1i1p1 |
| MIROC5  MIROC-ESM  MIROC-ESM-CHEM | JAMSTEC, Japan | r1i1p1 |
| MPI-ESM-MR | MPI-M, Germany | r1i1p1 |
| MRI-CGCM3  MRI-ESM1 | MRI, Japan | r1i1p1 |
| NorESM1-M | NCC, Norway | r1i1p1 |

| **CMIP6 Model** | **Institute, Country** | **Ensemble** |
| --- | --- | --- |
| ACCESS-CM2**^1,2^** | CSIRO, Australia | r1i1p1f1 |
| AWI-CM-1-1-MR**^3,4^** | AWI, Germany | r1i1p1f1 |
| CanESM5**^5,6^** | CCCMA, Canada | r1i1p1f1 |
| CESM2**^7,8^** | NCAR, USA | r1i1p1f1 |
| CIESM**^9,10^** | Tsinghua University, China | r1i1p1f1 |
| CMCC-CM2-SR5**^11,12^** | CMCC, Italy | r1i1p1f1 |
| CMCC-ESM2**^13,14^** |  |  |
| CNRM-CM6-1**^15,16^**  CNRM-ESM2-1**^17,18^** | CNRM, France | **r1i1p1f2** |
| E3SM-1-1**^19,20^** | DOE, USA | r1i1p1f1 |
| EC-Earth3**^21,22^**  EC-Earth3-CC**^23,24^**  EC-Earth3-Veg**^25,26^**  EC-Earth3-Veg-LR**^27,28^** | Europe-wide consortium | r1i1p1f1 |
| FGOALS-f3-L**^29,30^**  FGOALS-g3**^31,32^** | CAS, China | r1i1p1f1 |
| FIO-ESM-2-0**^33,34^** | FIO, China | r1i1p1f1 |
| GFDL-CM4**^35,36^** | NOAA-GFDL, USA | r1i1p1f1 |
| HadGEM3-GC31-LL**^37,38^**  HadGEM3-GC31-MM**^39,40^** | MOHC, UK | **r1i1p1f3** |
| KACE-1-0-G4**^41,42^** | NIMS-KMA, Korea | r1i1p1f1 |
| KIOST-ESM**^43,44^** | KIOST, Korea | r1i1p1f1 |
| MCM-UA-1-0**^45,46^** | UA, USA | **r1i1p1f2** |
| MIROC6**^47,48^** | JAMSTEC, Japan | r1i1p1f1 |
| MPI-ESM1-2-HR**^49,50^** | MPI-M, Germany | r1i1p1f1 |
| MRI-ESM2-0**^51,52^** | MRI, Japan | r1i1p1f1 |
| NorESM2-LM**^53,54^**  NorESM2-MM**^55,56^** | NCC, Norway | r1i1p1f1 |
| TaiESM1**^57,58^** | AS-RCEC, Taiwan | r1i1p1f1 |
| UKESM1-0-LL**^59,60^** | MOHC, UK | **r1i1p1f2** |


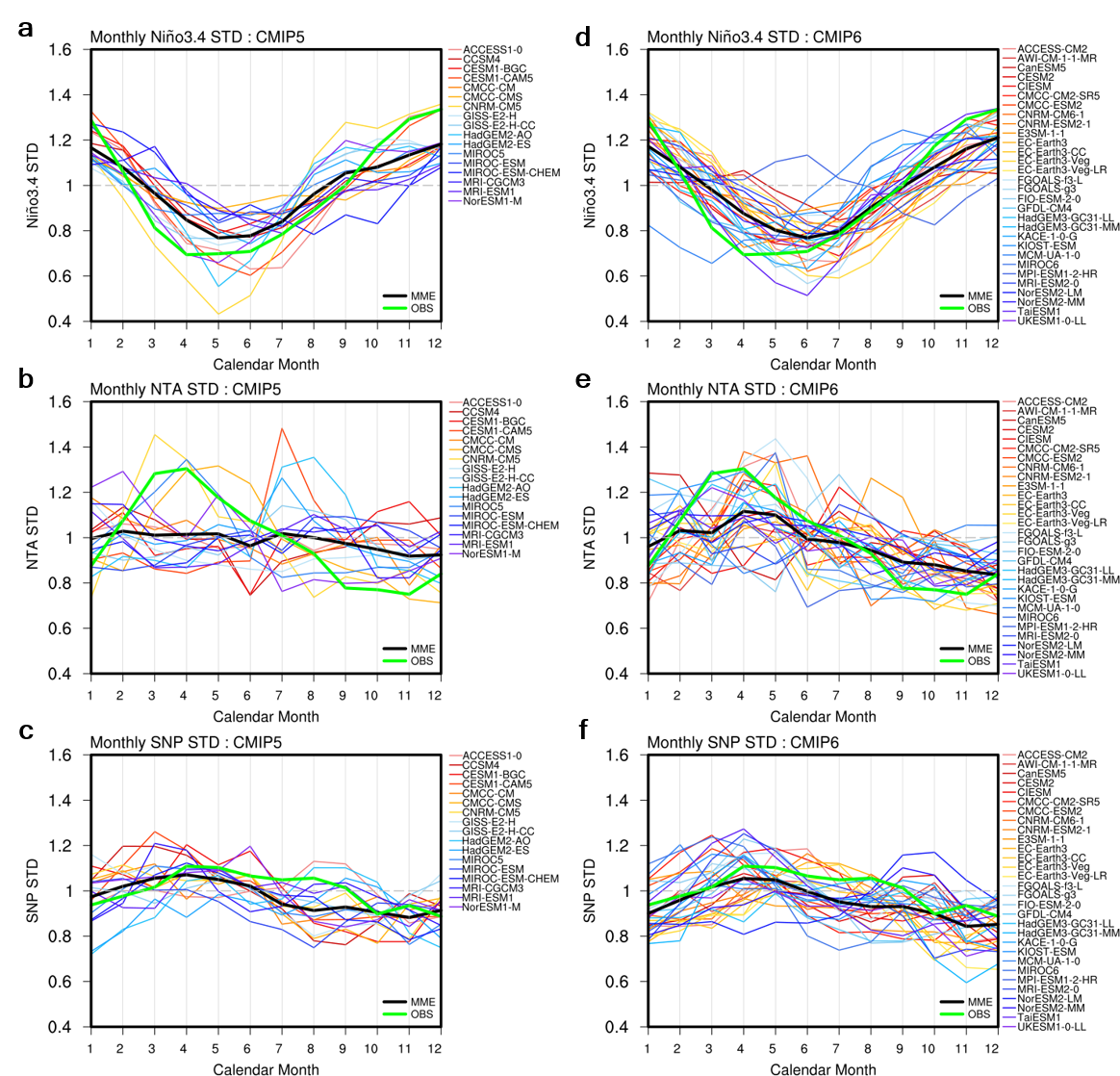


**Fig. S1.** **Seasonal variations of the ENSO, NTA, and SNP.** Monthly standard deviation (SD) of the (**a**) Niño3.4, (**b**) NTA, and (**c**) SNP indices in the 17 selected CMIP5 models. **d-f,** Same as **a-c** but for the 30 selected CMIP6 models. The black curves represent the multi-model ensemble (MME) values, and the green curves represent the observations. To focus on the seasonal variation, the values are normalized by the total respective SD of the indices using all months.


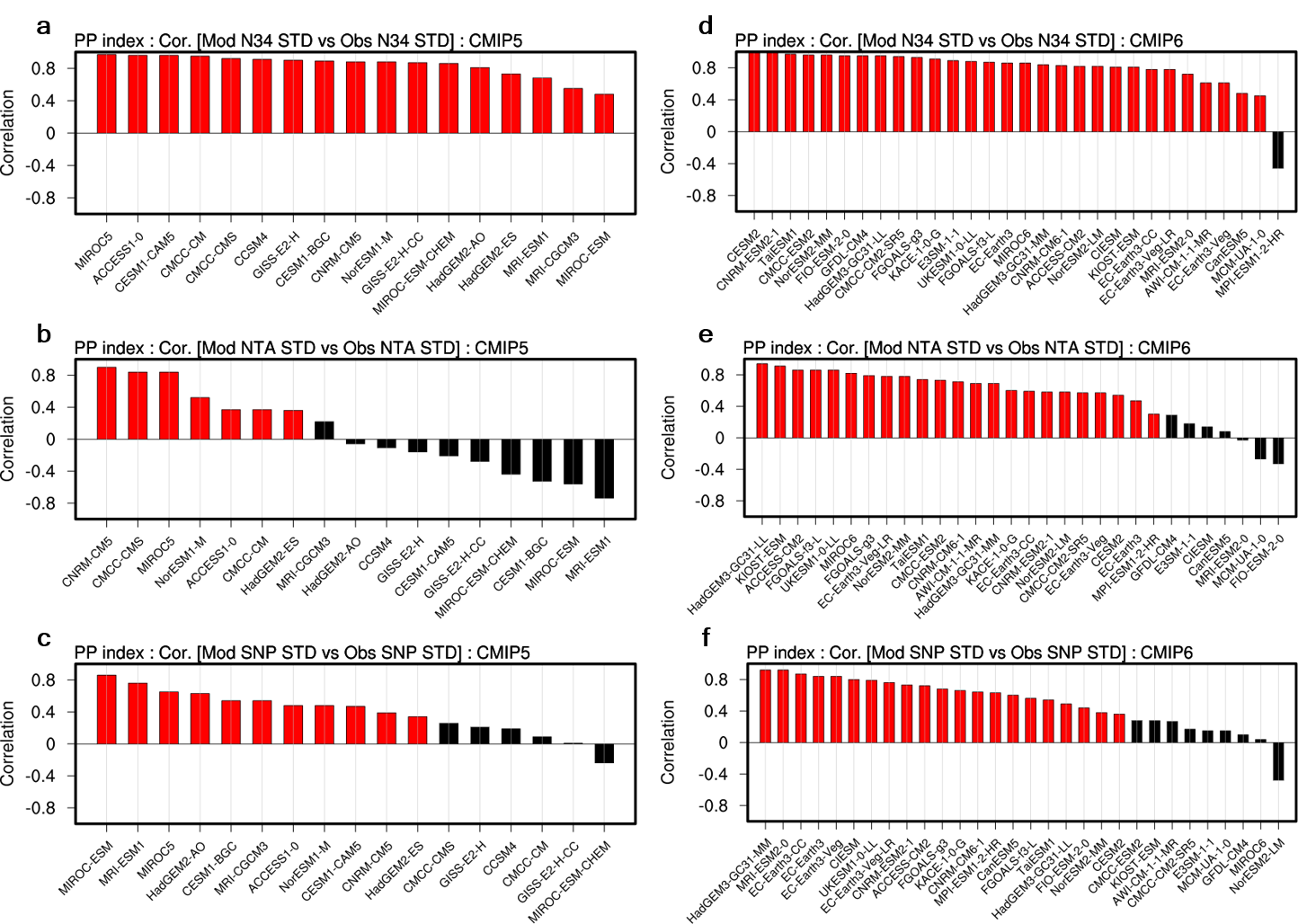


**Fig. S2. Evaluation of model’s performance in terms of ENSO, NTA, and SNP phase-locking.** Phase-locking Performance (PP) index of the 17 selected CMIP5 models for the (**a**) Niño3.4, (**b**) NTA, and (**c**) SNP indices, which is defined as the correlation coefficient between the observed and simulated monthly standard deviations of Niño3.4, NTA, and SNP, respectively. **d-f,** Same as **a-c** but for the selected 30 CMIP6 models. The red bars represent correlation coefficients above 0.3.


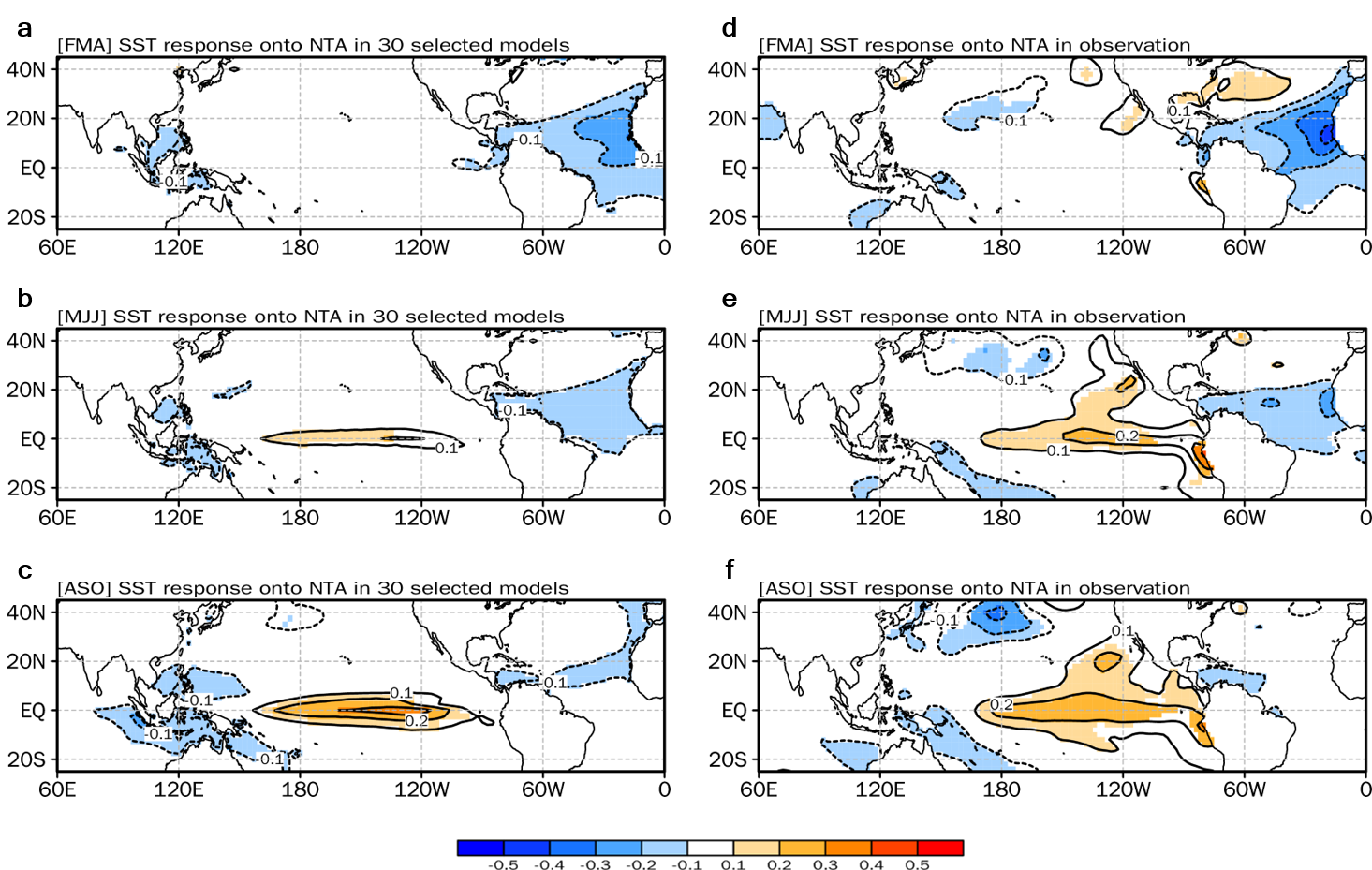


**Fig. S3. Modeled and observed time evolution of the NTA-induced SST anomalies.** Lag regression of SST (°C·s.d.^−1^; contours and shading) anomalies onto the FMA0 NTA SST during the FMA0, MJJ0, and ASO0 seasons based on the multi-model averaged value of the 30 selected CMIP6 models (**a-c**) and observations (**d-f**) under the present-day climate (1951-1999), respectively. Shading is shown for features with more than the 95% confidence level in **d-f** or the most robust features of the ensemble where the mean exceeds 1.0 s.d. in **a-c**. The color bar represents the regression coefficients (°C·s.d.^−1^). The regressed coefficients are multiplied by −1 for the NTA SST.


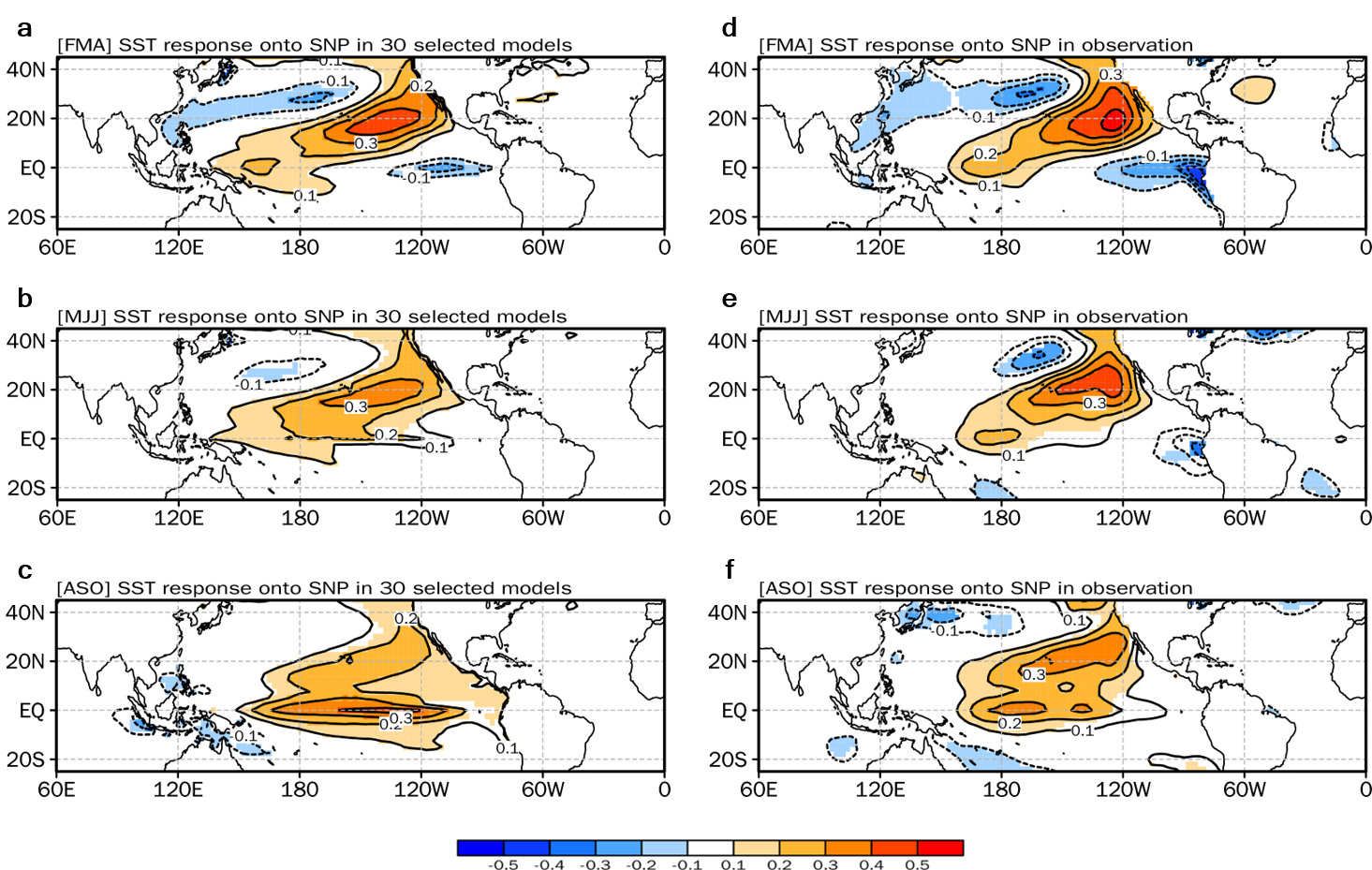


**Fig. S4. Modeled and observed time evolution of the SNP-induced SST anomalies.** Lag regression of SST (°C·s.d.^−1^; contours and shading) anomalies onto the FMA0 SNP SST during the FMA0, MJJ0, and ASO0 seasons based on the multi-model averaged value of the 30 selected CMIP6 models (**a-c**) and observations (**d-f**) under the present-day climate (1951-1999), respectively. Shading is shown for features with more than the 95% confidence level in **d-f** or the most robust features of the ensemble where the mean exceeds 1.0 s.d. in **a-c**. The color bar represents the regression coefficients (°C·s.d.^−1^).


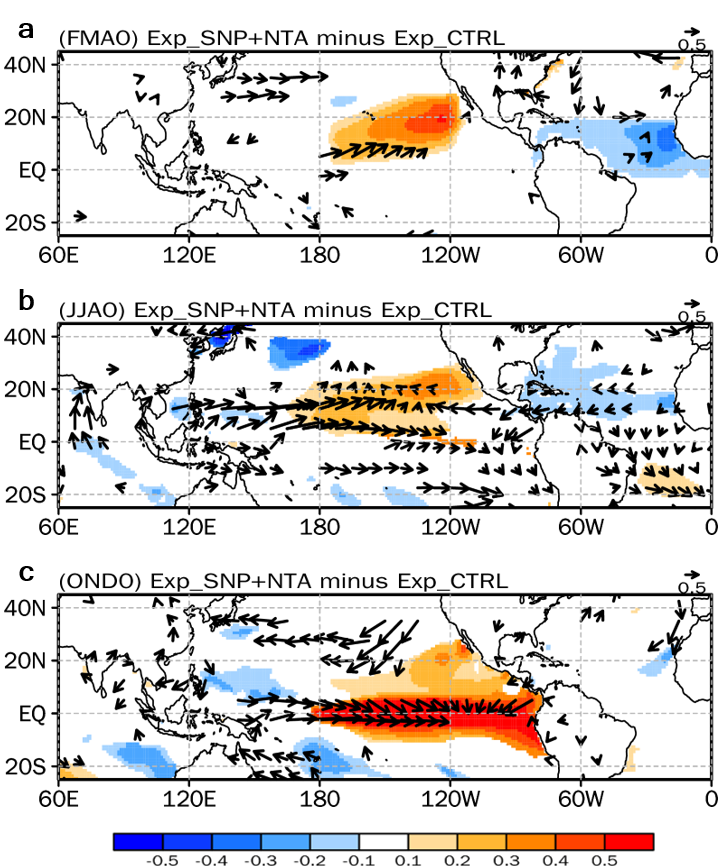


**Fig. S5. Idealized partially-coupled model experiments.** Changes in the SST (shading, °C) and 925hPa wind (vector, m·s^−1^) anomalies in the north tropical Atlantic (NTA) and subtropical North Pacific (SNP) experiment (Exp_NTA+SNP) compared to the control experiment (Exp_CTRL) under a pre-industrial CO_2_ concentration (284 ppm) during the FMA0 (**a**), JJA0 (**b**), and OND0 (**c**) seasons. The shading and vector denote regions where the statistical significance is above the 90% confidence level.


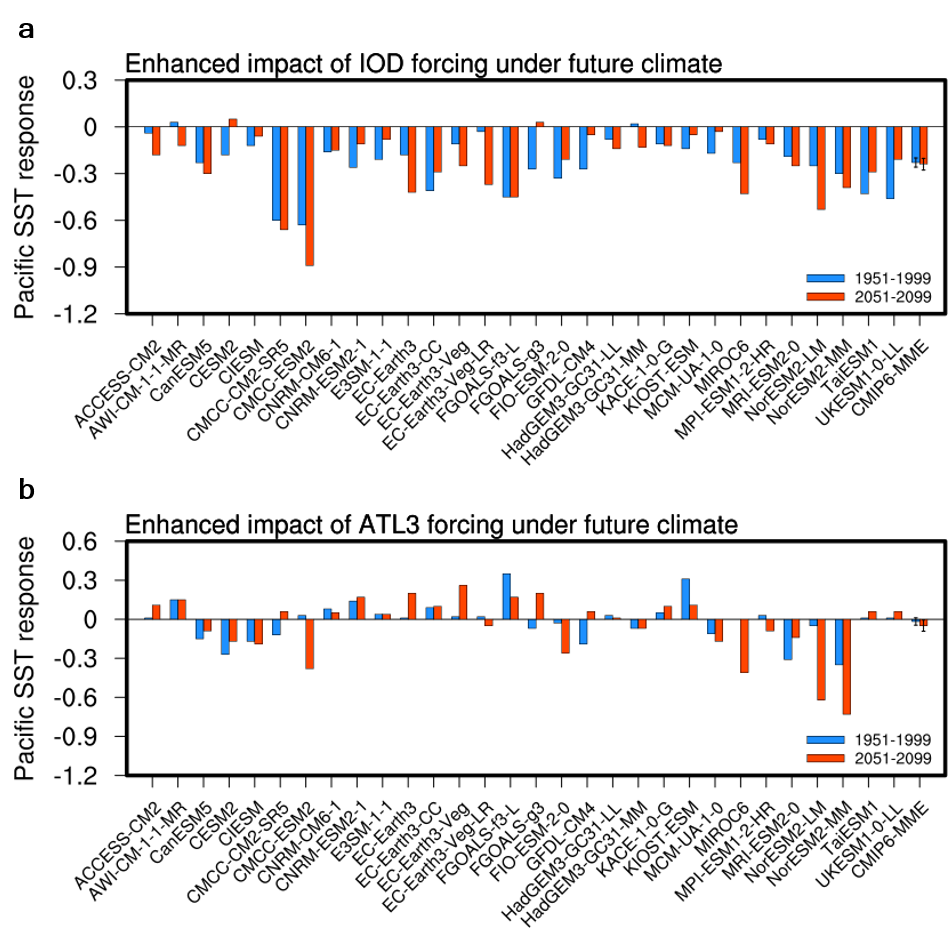


**Fig. S6. Negligible impacts of the equatorial Indian or Atlantic ENSO precursors under greenhouse warming. a,** Comparison of the D0JF1 equatorial Pacific SST response (°C·s.d.^−1^) to the Indian Ocean Dipole during the preceding boreal fall (SON–1) under the present-day climate (1951–1999; blue bars) and future climate (2051–2099; red bars) in the 30 selected CMIP6 models. The multi-model averaged value and corresponding error bars for the 30 selected CMIP6 models (labeled CMIP6-MME) are also shown. **b,** Same as **a** but for the Atlantic Niño/Niña during the boreal summer (JJA0). Error bars are calculated as 1.0 s.d. of 10,000 inter-realizations of a bootstrap method (see Methods for details on the bootstrap test).


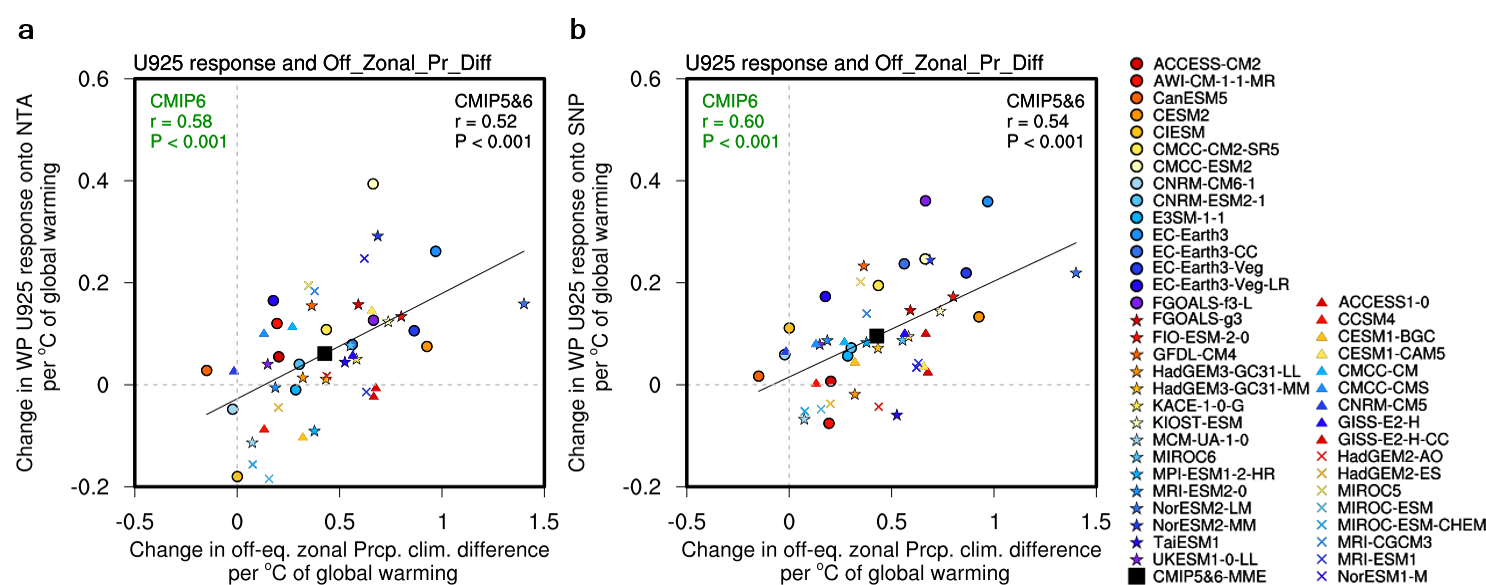


**Fig. S7. Stronger NTA-, and SNP-related westerly anomalies associated with the wetter mean state change over the off-equatorial eastern Pacific. a,b,** Inter-model relationship between changes (future minus present day) of the FMAMJJASO0 equatorial western–central Pacific 925 hPa zonal wind (5° S–5° N, 140° E–160° W) response ($y$ axis; m·s^-1^·s.d.^-1^·°C^-1^) for the FMA0 NTA SST (**a**) and SNP SST (**b**) with the FMAMJJASO0 off-equatorial zonal precipitation climatology difference [(150°–90° W, 2° S–8° N) minus (130°–150° E, 2° S–8° N)] ($x$ axis; mm·day^−1^· °C^−1^). For the models with available data, the CMIP5 models are denoted by triangles and crosses and the CMIP6 models are denoted by dots and stars (see CMIP5/6 data in Methods). A linear fit is displayed along with the correlation coefficient $r$ and $P$ value based on the 30 selected CMIP6 models (green text) and the 47 selected CMIP5/6 models (black line and black text). The multi-model averaged value for the CMIP5/6 models (labeled CMIP5&6-MME) is also denoted by black squares. The changes in each model are scaled by the corresponding increase in the global mean temperature. The regressed coefficients are multiplied by −1 for the NTA SST.


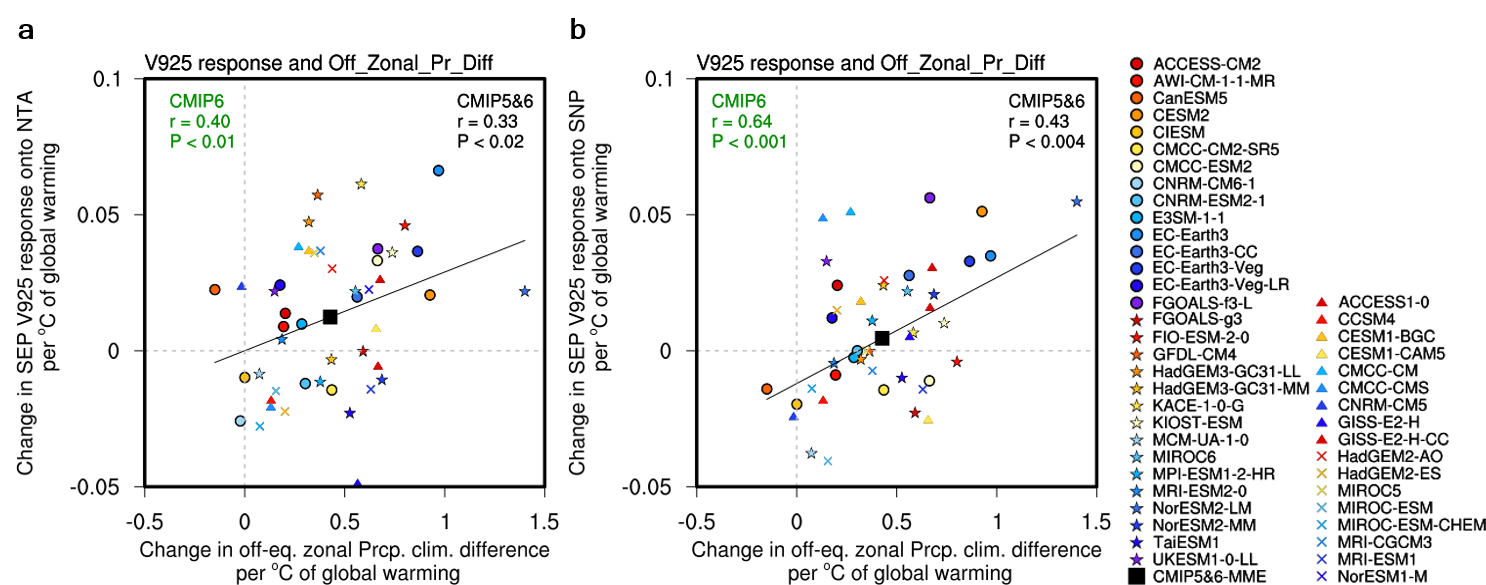


**Fig. S8. Stronger NTA-, and SNP-related northerly anomalies associated with the wetter mean state change over the off-equatorial eastern Pacific. a,b,** Inter-model relationship between the changes (future minus present day) of the FMAMJJASO0 subtropical eastern Pacific 925 hPa meridional wind (10°–25° N, 150°–120° W) response ($y$ axis; m·s^−1^·s.d.^−1^·°C^−1^) for the FMA0 NTA SST (**a**) and SNP SST (**b**) with the FMAMJJASO0 off-equatorial zonal precipitation climatology difference [(150°–90° W, 2° S–8° N) minus (130°–150° E, 2° S–8° N)] ($x$ axis; mm·day^−1^· °C^−1^). For the models with available data, the CMIP5 models are denoted by triangles and crosses and the CMIP6 models are denoted by dots and stars (see CMIP5/6 data in Methods). A linear fit is displayed along with the correlation coefficient $r$ and $P$ value based on the 30 selected CMIP6 models (green text) and the 47 selected CMIP5/6 models (black line and black text). The multi-model averaged value for the CMIP5/6 models (labeled CMIP5&6-MME) is also denoted by black squares. The changes in each model are scaled by the corresponding increase in the global mean temperature. The regressed coefficients are multiplied by −1 for the NTA SST.


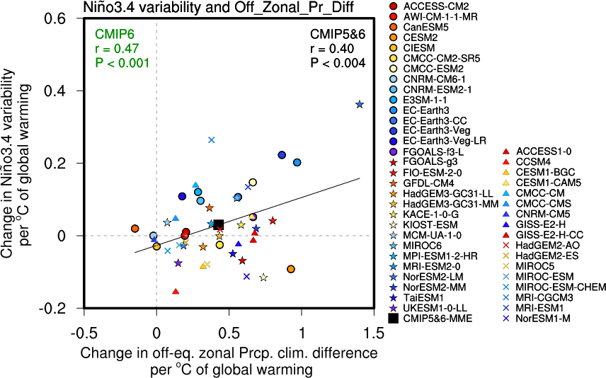


**Fig. S9. Impact of the wetter mean state over the off-equatorial eastern Pacific on ENSO amplitude under greenhouse warming.** Inter-model relationship between the change (future minus present day) of the D0JF1 Niño3.4 (5° S–5° N, 170°–120° W) SST variability ($y$ axis; s.d.·°C^−1^) with the FMAMJJASO0 off-equatorial zonal precipitation climatology difference [(150°–90° W, 2° S–8° N) minus (130°–150° E, 2° S–8° N)] ($x$ axis; mm·day^−1^· °C^−1^). For the models with available data, the CMIP5 models are denoted by triangles and crosses and the CMIP6 models are denoted by dots and stars (see CMIP5/6 data in Methods). A linear fit is displayed along with the correlation coefficient $r$ and $P$ value based on the 30 selected CMIP6 models (green text) and the 47 selected CMIP5/6 models (black line and black text). The multi-model averaged value for the CMIP5/6 models (labeled CMIP5&6-MME) is also denoted by black squares. The changes in each model are scaled by the corresponding increase in the global mean temperature.


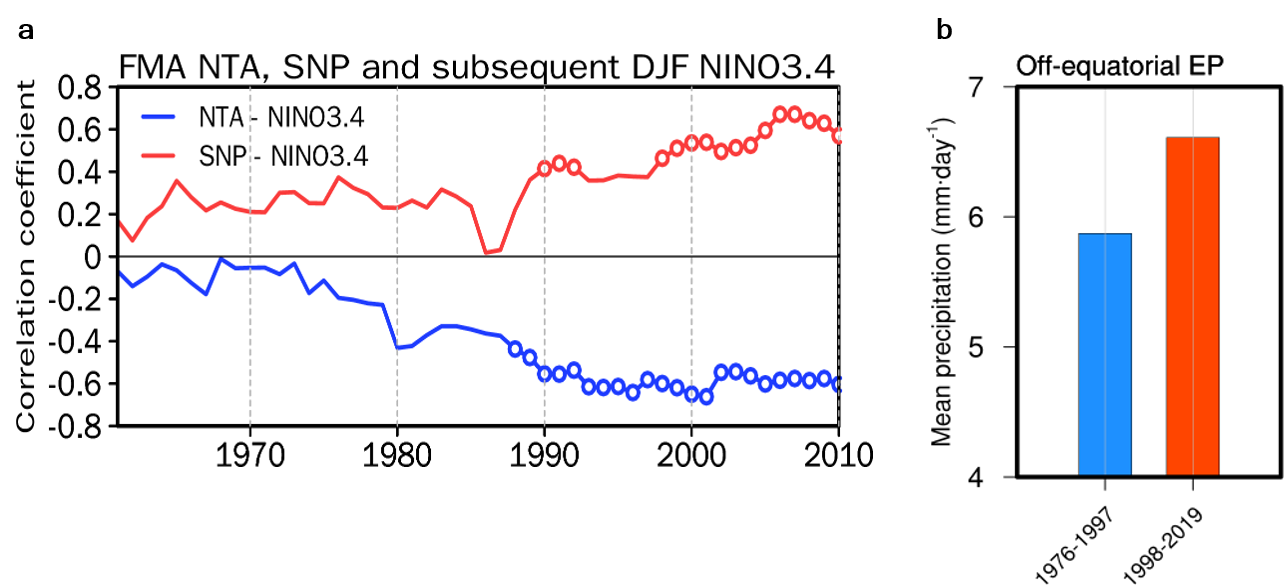


**Fig. S10. Enhanced NTA−ENSO and SNP−ENSO relationship with the wetter mean state over the off-equatorial eastern Pacific after the 2000s. a,** 21-yr moving correlation coefficients between the NTA and SNP index during the boreal spring season (FMA0), and the subsequent year’s Nino3.4 index during the boreal winter season (D0JF1) (blue line: NTA vs. Nino3.4; red line: SNP vs. Nino3.4) from 1951-2020 in observations. The x-axis indicates the middle year in the 21-yr moving window (e.g., 1999 indicates the correlation coefficient from 1989−2009). The open circles represent correlation coefficients that are statistically significant at the 95% confidence level. **b,** Area-averaged mean precipitation (mm·day^−1^) over the off-equatorial eastern Pacific (150°–90° W, 2° S–8° N) during the spring-to-fall seasons (February to October) for 1976–1997 (*blue bar*) and 1998–2019 (*red bar*) in observations.


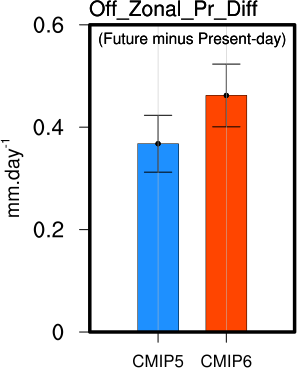


**Fig. S11. Changes in the Off_Zonal_Pr_Diff.** Changes (future minus present day) in the Off_Zonal_Pr_Diff are shown based on the selected 17 CMIP5 and 30 CMIP6 models, represented by blue and red bars, respectively. Error bars are calculated as 1.0 s.d. of 10,000 inter-realizations of a bootstrap method (see Methods for details on the bootstrap test). The CMIP6 models tend to simulate a wetter mean state over the off-equatorial eastern Pacific compared to the CMIP5 models under greenhouse warming.

**Supplementary References**

1. Dix, M. et al. *CSIRO-ARCCSS ACCESS-CM2 model output prepared for CMIP6 CMIP Historical* Version 20220519 (Earth System Grid Federation, 2019); https://doi.org/10.22033/ESGF/CMIP6.4271

2. Dix, M. et al. *CSIRO-ARCCSS ACCESS-CM2 model output prepared for CMIP6 ScenarioMIP ssp585* Version 20220519 (Earth System Grid Federation, 2019); https://doi.org/10.22033/ESGF/CMIP6.4332

3. Semmler, T. et al. *AWI AWI-CM1.1MR Model Output Prepared for CMIP6 CMIP Historical* Version 20220519 (Earth System Grid Federation, 2018); https://doi.org/10.22033/ESGF/CMIP6.2686

4. Semmler, T. et al. *AWI AWI-CM1.1MR Model Output Prepared for CMIP6 ScenarioMIP ssp585* Version 20220519 (Earth System Grid Federation, 2019); https://doi.org/10.22033/ESGF/CMIP6.2817

5. Swart, N. C. et al. *CCCma CanESM5 Model Output Prepared for CMIP6 CMIP Historical* Version 20220519 (Earth System Grid Federation, 2019); https://doi.org/10.22033/ESGF/CMIP6.3610

6. Swart, N. C. et al. *CCCma CanESM5 Model Output Prepared for CMIP6 ScenarioMIP ssp585* Version 20220519 (Earth System Grid Federation, 2019); https://doi.org/10.22033/ESGF/CMIP6.3696

7. Danabasoglu, G. *NCAR CESM2 Model Output Prepared for CMIP6 CMIP Historical* Version 20220519 (Earth System Grid Federation, 2019); https://doi.org/10.22033/ESGF/CMIP6.7627

8. Danabasoglu, G. *NCAR CESM2 Model Output Prepared for CMIP6 ScenarioMIP ssp585* Version 20220519 (Earth System Grid Federation, 2019); https://doi.org/10.22033/ESGF/CMIP6.7768

9. Huang, W. *THU CIESM Model Output Prepared for CMIP6 CMIP Historical* Version 20220519 (Earth System Grid Federation, 2019); https://doi.org/10.22033/ESGF/CMIP6.8843

10. Huang, W. *THU CIESM Model Output Prepared for CMIP6 ScenarioMIP ssp585* Version 20220519 (Earth System Grid Federation, 2020); https://doi.org/10.22033/ESGF/CMIP6.8863

11. Lovato, T. & Peano, D. *CMCC CMCC-CM2-SR5 Model Output Prepared for CMIP6 CMIP Historical* Version 20220519 (Earth System Grid Federation, 2020); https://doi.org/10.22033/ESGF/CMIP6.3825

12. Lovato, T. & Peano, D. *CMCC CMCC-CM2-SR5 Model Output Prepared for CMIP6 ScenarioMIP ssp585* Version 20220519 (Earth System Grid Federation, 2020); https://doi.org/10.22033/ESGF/CMIP6.3896

13. Lovato, T. et al. *CMCC CMCC-ESM2 Model Output Prepared for CMIP6 CMIP Historical* Version 20220519 (Earth System Grid Federation, 2021); https://doi.org/10.22033/ESGF/CMIP6.13195

14. Lovato, T. et al. *CMCC CMCC-ESM2 Model Output Prepared for CMIP6 ScenarioMIP* Version 20220519 (Earth System Grid Federation, 2021); https://doi.org/10.22033/ESGF/CMIP6.13168

15. Voldoire, A. *CMIP6 simulations of the CNRM-CERFACS based on CNRM-CM6-1 model for CMIP experiment historical* Version 20220519 (Earth System Grid Federation, 2018); https://doi.org/10.22033/ESGF/CMIP6.4066

16. Voldoire, A. *CNRM-CERFACS CNRM-CM6-1 Model Output Prepared for CMIP6 ScenarioMIP ssp585* Version 20220519 (Earth System Grid Federation, 2019); https://doi.org/10.22033/ESGF/CMIP6.4224

17. Seferian, R. *CNRM-CERFACS CNRM-ESM2-1 Model Output Prepared for CMIP6 CMIP Historical* Version 20220519 (Earth System Grid Federation, 2018); https://doi.org/10.22033/ESGF/CMIP6.4068

18. Voldoire, A. *CNRM-CERFACS CNRM-ESM2-1 Model Output Prepared for CMIP6 ScenarioMIP ssp585* Version 20220519 (Earth System Grid Federation, 2019); https://doi.org/10.22033/ESGF/CMIP6.4226

19. Bader, D. C., Leung, R., Taylor, M. & McCoy, R. B. *E3SM-Project E3SM1.1 Model Output Prepared for CMIP6 CMIP Historical* Version 20220519 (Earth System Grid Federation, 2019); https://doi.org/10.22033/ESGF/CMIP6.11485

20. Bader, D. C., Leung, R., Taylor, M. & McCoy, R. B. *E3SM-Project E3SM1.1 Model Output Prepared for CMIP6 ScenarioMIP ssp585* Version 20220519 (Earth System Grid Federation, 2020); https://doi.org/10.22033/ESGF/CMIP6.15179

21. EC-Earth Consortium (EC-Earth). *EC-Earth-Consortium EC-Earth3 Model Output Prepared for CMIP6 CMIP Historical* Version 20220519 (Earth System Grid Federation, 2019); https://doi.org/10.22033/ESGF/CMIP6.4700

22. EC-Earth Consortium (EC-Earth). *EC-Earth-Consortium EC-Earth3 Model Output Prepared for CMIP6 ScenarioMIP ssp585* Version 20220519 (Earth System Grid Federation, 2019); https://doi.org/10.22033/ESGF/CMIP6.4912

23. EC-Earth Consortium (EC-Earth). *EC-Earth-Consortium EC-Earth-3-CC Model Output Prepared for CMIP6 CMIP Historical* Version 20220519 (Earth System Grid Federation, 2021); https://doi.org/10.22033/ESGF/CMIP6.4702

24. EC-Earth Consortium (EC-Earth). *EC-Earth-Consortium EC-Earth3-CC Model Output Prepared for CMIP6 ScenarioMIP ssp585* Version 20220519 (Earth System Grid Federation, 2021); https://doi.org/10.22033/ESGF/CMIP6.15636

25. EC-Earth Consortium (EC-Earth). *EC-Earth-Consortium EC-Earth3-Veg Model Output Prepared for CMIP6 CMIP Historical* Version 20220519 (Earth System Grid Federation, 2019); https://doi.org/10.22033/ESGF/CMIP6.4706

26. EC-Earth Consortium (EC-Earth). *EC-Earth-Consortium EC-Earth3-Veg Model Output Prepared for CMIP6 ScenarioMIP ssp585* Version 20220519 (Earth System Grid Federation, 2019); https://doi.org/10.22033/ESGF/CMIP6.4914

27. EC-Earth Consortium (EC-Earth). *EC-Earth-Consortium EC-Earth3-Veg-LR Model Output Prepared for CMIP6 CMIP Historical* Version 20220519 (Earth System Grid Federation, 2020); https://doi.org/10.22033/ESGF/CMIP6.4707

28. EC-Earth Consortium (EC-Earth). *EC-Earth-Consortium EC-Earth3-Veg-LR Model Output Prepared for CMIP6 ScenarioMIP ssp585* Version 20220519 (Earth System Grid Federation, 2020); https://doi.org/10.22033/ESGF/CMIP6.4915

29. Yu, Y. *CAS FGOALS-f3-L Model Output Prepared for CMIP6 CMIP Historical* Version 20220519 (Earth System Grid Federation, 2019); https://doi.org/10.22033/ESGF/CMIP6.3355

30. Yu, Y. *CAS FGOALS-f3-L Model Output Prepared for CMIP6 ScenarioMIP ssp585* Version 20220519 (Earth System Grid Federation, 2019); https://doi.org/10.22033/ESGF/CMIP6.3502

31. Li, L. *CAS FGOALS-g3 Model Output Prepared for CMIP6 CMIP Historical* Version 20220519 (Earth System Grid Federation, 2019); https://doi.org/10.22033/ESGF/CMIP6.3356

32. Li, L. *CAS FGOALS-g3 Model Output Prepared for CMIP6 ScenarioMIP ssp585* Version 20220519 (Earth System Grid Federation, 2019); https://doi.org/10.22033/ESGF/CMIP6.3503

33. Song, Z. et al. *FIO-QLNM FIO-ESM2.0 Model Output Prepared for CMIP6 CMIP Historical* Version 20220519 (Earth System Grid Federation, 2019); https://doi.org/10.22033/ESGF/CMIP6.9199

34. Song, Z. et al. *FIO-QLNM FIO-ESM2.0 Model Output Prepared for CMIP6 ScenarioMIP ssp585* Version 20220519 (Earth System Grid Federation, 2019); https://doi.org/10.22033/ESGF/CMIP6.9214

35. Guo, H. et al. *NOAA-GFDL GFDL-CM4 Model Output Prepared for CMIP6 CMIP Historical* Version 20220519 (Earth System Grid Federation, 2018); https://doi.org/10.22033/ESGF/CMIP6.8594

36. Guo, H. et al. *NOAA-GFDL GFDL-CM4 Model Output Prepared for CMIP6 ScenarioMIP ssp585* Version 20220519 (Earth System Grid Federation, 2018); https://doi.org/10.22033/ESGF/CMIP6.9268

37. Ridley, J. et al. *MOHC HadGEM3-GC31-LL Model Output Prepared for CMIP6 CMIP Historical* Version 20220519 (Earth System Grid Federation, 2019); https://doi.org/10.22033/ESGF/CMIP6.6109

38. Good, P. *MOHC HadGEM3-GC31-LL Model Output Prepared for CMIP6 ScenarioMIP ssp585* Version 20220519 (Earth System Grid Federation, 2020); https://doi.org/10.22033/ESGF/CMIP6.10901

39. Ridley, J. et al. *MOHC HadGEM3-GC31-MM Model Output Prepared for CMIP6 CMIP Historical* Version 20220519 (Earth System Grid Federation, 2019); https://doi.org/10.22033/ESGF/CMIP6.6112

40. Jackson, L. *MOHC HadGEM3-GC31-MM Model Output Prepared for CMIP6 ScenarioMIP ssp585* Version 20220519 (Earth System Grid Federation, 2020); <https://doi.org/10.22033/ESGF/CMIP6.10902>

41. Byun, Y. H. et al. *NIMS-KMA KACE1.0-G Model Output Prepared for CMIP6 CMIP Historical* Version 20220519 (Earth System Grid Federation, 2019); https://doi.org/10.22033/ESGF/CMIP6.8378

42. Byun, Y. H. et al. *NIMS-KMA KACE1.0-G Model Output Prepared for CMIP6 ScenarioMIP ssp585* Version 20220519 (Earth System Grid Federation, 2019); https://doi.org/10.22033/ESGF/CMIP6.8456

43. Kim, Y. H. et al. *KIOST KIOST-ESM Model Output Prepared for CMIP6 CMIP Historical* Version 20220519 (Earth System Grid Federation, 2019); https://doi.org/10.22033/ESGF/CMIP6.5296

44. Kim, Y. H. et al. *KIOST KIOST-ESM Model Output Prepared for CMIP6 ScenarioMIP ssp585* Version 20220519 (Earth System Grid Federation, 2019); https://doi.org/10.22033/ESGF/CMIP6.11249

45. Stouffer, R. *UA MCM-UA-1-0 Model Output Prepared for CMIP6 CMIP Historical* Version 20220519 (Earth System Grid Federation, 2019); https://doi.org/10.22033/ESGF/CMIP6.8888

46. Stouffer, R. *UA MCM-UA-1-0 Model Output Prepared for CMIP6 ScenarioMIP ssp585* Version 20220519 (Earth System Grid Federation, 2019); https://doi.org/10.22033/ESGF/CMIP6.13901

47. Tatebe, H. & Watanabe, M. *MIROC MIROC6 Model Output Prepared for CMIP6 CMIP Historical* Version 20220519 (Earth System Grid Federation, 2018); https://doi.org/10.22033/ESGF/CMIP6.5603

48. Shiogama, H. et al. *MIROC MIROC6 Model Output Prepared for CMIP6 ScenarioMIP ssp585* Version 20220519 (Earth System Grid Federation, 2019); https://doi.org/10.22033/ESGF/CMIP6.5771

49. Jungclaus, J. et al. *MPI-M MPI-ESM1.2-HR Model Output Prepared for CMIP6 CMIP Historical* Version 20220519 (Earth System Grid Federation, 2019); https://doi.org/10.22033/ESGF/CMIP6.6594

50. Steger, C. et al. *DWD MPI-ESM1.2-HR Model Output Prepared for CMIP6 ScenarioMIP ssp585* Version 20220519 (Earth System Grid Federation, 2019); https://doi.org/10.22033/ESGF/CMIP6.4479

51. Yukimoto, S. et al. *MRI MRI-ESM2.0 Model Output Prepared for CMIP6 CMIP Historical* Version 20220519 (Earth System Grid Federation, 2019); https://doi.org/10.22033/ESGF/CMIP6.6842

52. Yukimoto, S. et al. *MRI MRI-ESM2.0 Model Output Prepared for CMIP6 ScenarioMIP ssp585* Version 20220519 (Earth System Grid Federation, 2019); https://doi.org/10.22033/ESGF/CMIP6.6929

53. Seland, Ø. et al. *NCC NorESM2-LM Model Output Prepared for CMIP6 CMIP Historical* Version 20220519 (Earth System Grid Federation, 2019); https://doi.org/10.22033/ESGF/CMIP6.8036

54. Seland, Ø. et al. *NCC NorESM2-LM Model Output Prepared for CMIP6 ScenarioMIP ssp585* Version 20220519 (Earth System Grid Federation, 2019); https://doi.org/10.22033/ESGF/CMIP6.8319

55. Bentsen, M. et al. *NCC NorESM2-MM Model Output Prepared for CMIP6 CMIP Historical* Version 20220519 17 (Earth System Grid Federation, 2019); https://doi.org/10.22033/ESGF/CMIP6.8040

56. Bentsen, M. et al. *NCC NorESM2-MM Model Output Prepared for CMIP6 ScenarioMIP ssp585* Version 20220519 (Earth System Grid Federation, 2019); <https://doi.org/10.22033/ESGF/CMIP6.8321>

57. Lee, W. & Liang, H.C. *AS-RCEC TaiESM1.0 Model Output Prepared for CMIP6 CMIP Historical* Version 20220519 17 (Earth System Grid Federation, 2020); https://doi.org/10.22033/ESGF/CMIP6.9755

58. Lee, W. & Liang, H.C. *AS-RCEC TaiESM1.0 Model Output Prepared for CMIP6 ScenarioMIP ssp585* Version 20220519 (Earth System Grid Federation, 2020); [https://doi.org/10.22033/ESGF/CMIP6.9823](https://doi.org/10.22033/ESGF/CMIP6.8321)

59. Tang, Y. et al. *MOHC UKESM1.0-LL Model Output Prepared for CMIP6 CMIP Historical* Version 20220519 (Earth System Grid Federation, 2019); https://doi.org/10.22033/ESGF/CMIP6.6113

60. Good, P. et al. *MOHC UKESM1.0-LL Model Output Prepared for CMIP6 ScenarioMIP ssp585* Version 20220519 (Earth System Grid Federation, 2019); https://doi.org/10.22033/ESGF/CMIP6.6405
